# Melanoma Brain Metastasis: Mechanisms, Models, and Medicine

**DOI:** 10.3390/ijms17091468

**Published:** 2016-09-02

**Authors:** David A. Kircher, Mark R. Silvis, Joseph H. Cho, Sheri L. Holmen

**Affiliations:** 1 Department of Oncological Sciences, University of Utah Health Sciences Center, Salt Lake City, UT 84112, USA; david.kircher@hci.utah.edu (D.A.K.); joe.cho@hsc.utah.edu (J.H.C.); 2 Department of Surgery, University of Utah Health Sciences Center, Salt Lake City, UT 84112, USA; mark.silvis@hci.utah.edu; 3 Huntsman Cancer Institute, University of Utah Health Sciences Center, Salt Lake City, UT 84112, USA

**Keywords:** melanoma, brain metastases, mechanisms, models, clinical trials

## Abstract

The development of brain metastases in patients with advanced stage melanoma is common, but the molecular mechanisms responsible for their development are poorly understood. Melanoma brain metastases cause significant morbidity and mortality and confer a poor prognosis; traditional therapies including whole brain radiation, stereotactic radiotherapy, or chemotherapy yield only modest increases in overall survival (OS) for these patients. While recently approved therapies have significantly improved OS in melanoma patients, only a small number of studies have investigated their efficacy in patients with brain metastases. Preliminary data suggest that some responses have been observed in intracranial lesions, which has sparked new clinical trials designed to evaluate the efficacy in melanoma patients with brain metastases. Simultaneously, recent advances in our understanding of the mechanisms of melanoma cell dissemination to the brain have revealed novel and potentially therapeutic targets. In this review, we provide an overview of newly discovered mechanisms of melanoma spread to the brain, discuss preclinical models that are being used to further our understanding of this deadly disease and provide an update of the current clinical trials for melanoma patients with brain metastases.

## 1. Tumor Cell Metastasis

Metastasis is a complex, multi-step process resulting in the spread of tumor cells from a primary lesion to a disparate organ or organs within the body, that results in increased patient morbidity and mortality [1]. Metastatic cells acquire unique characteristics that enable them to proliferate and migrate, invade the local normal tissue, intravasate through the basement membrane into blood or lymphatic vessels, survive during circulation through the blood stream or lymphatic system, arrest at distant sites, extravasate into the surrounding tissue, and proliferate by inducing angiogenesis [1]. In addition to cancer cell-autonomous phenotypes that enable this process to occur, the microenvironment of the metastatic site also provides pro-tumorigenic signals, innately, or in response to the metastasis itself, which promote survival, migration, and growth of disseminated tumor cells at secondary sites [2,3]. The brain microenvironment is a unique physiological niche due to its highly selective blood-brain barrier (BBB), high-energy consumption and nutritional demands, and immune privilege [4]. During the final stages of melanoma cell metastasis in the brain, circulating tumor cells adhere to the vasculature and bypass the BBB, the specialized multicellular layer that protects the brain, thereby establishing secondary neoplasms within the brain. Several lines of evidence suggest that metastatic melanoma cells are genetically evolved from their primary site predecessors and thus have acquired phenotypes that select for cerebrotropism [5,6,7]. In addition, the brain milieu provides necessary cues for survival, migration, proliferation, and angiogenesis, thus facilitating melanoma brain metastasis. The following review provides an overview of our current mechanistic understanding of melanoma brain metastasis formation, a more detailed overview of the role of the AKT/PI3K pathway in melanoma progression, an examination of models used to study these processes, and an update of current clinical trials for melanoma patients with brain metastases.

## 2. Mechanisms of Melanoma Brain Metastasis

**Figure 1:**
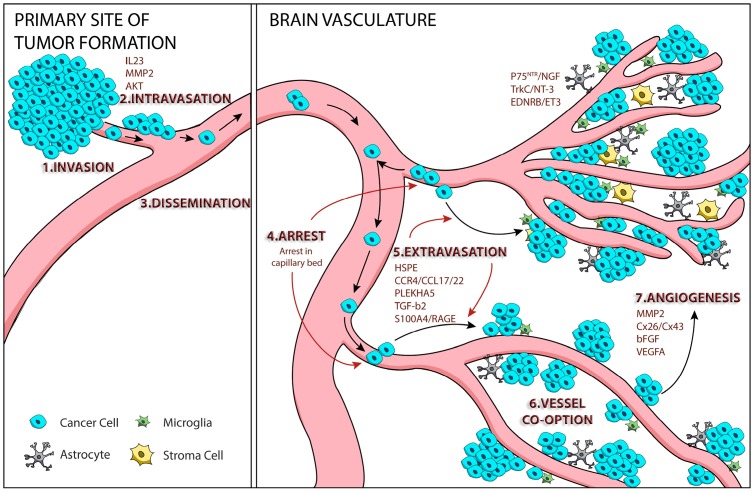
Steps and factors involved in melanoma metastasis to the brain.

Metastatic brain tumors are the most common intracranial neoplasm in adults [8]. To better understand the formation of brain metastases, the Winkler lab used a mouse with a chronic cranial window to track the fate of fluorescently labeled melanoma cells during their colonization of the mouse brain. They characterized the steps of the late metastatic cascade as: cell arrest at vascular branch points, early extravasation, persistent close contact to microvessels, perivascular growth by vessel co-option, and angiogenesis [9]. These steps provide a general framework in which to discuss the mechanisms underlying intracranial melanoma metastasis formation (Figure 1).

### 2.1. Melanoma Cell Arrest within the Brain Microvasculature

To address how passively diffusing, disseminated metastatic melanoma cells arrive at the brain microvasculature and establish a secondary neoplasm, Kienast et al. examined the specific intravascular location of metastases formation. They noted that movement of the injected cells through the blood vessels of the brain was initially halted as the diameter of the microvessels decreased to a size comparable with that of the cells, and especially at vascular branch points [9]. This suggests that disseminated metastatic melanoma cells can become physically lodged within brain capillaries. The pattern of blood flow throughout the body and mechanical limitations presented by the vasculature both influence metastatic establishment of cancer cells at distinct organs [10]. In addition to the physical impediment of cell mobility caused by microvessel size or bifurcation, it is well documented that metastatic melanoma cells also display increased adhesion to the brain endothelium, as compared to non-metastatic melanoma cells, or even metastatic breast cancer cells [11,12,13]. Several adhesion molecules that were originally described in leukocytes and platelets, including selectins, integrins, cell adhesion molecules, tetraspanins, and others, have been shown to mediate metastatic cell binding to the brain endothelium (reviewed in [14]); however, to what extent each of these adhesion molecules contributes to the colonization of the brain by melanoma cells specifically is either not known or poorly understood.

Kienast et al. also noted that arrested cells were able to evacuate from their initial resting place and subsequently relocate to another site within the brain vasculature [9]. This observation corroborates what other studies have shown—that disseminated melanoma cells can be directed to and/or selectively retained within the brain by external cues originating in the parenchymal milieu, such as chemokines or brain-derived ligands. Chemokine stimulation of their respective receptors is known to induce cellular responses of altered adhesion, migration, proliferation, and apoptosis [15]. Brain-derived signals promote melanoma cell adhesion to the intracranial vasculature and concomitantly foster melanoma metastases formation, which is the “seed and soil” hypothesis of cancer cell metastasis originally proposed by Paget in 1889 [16].

The role of chemokines and their cognate receptors in directing organ-specific melanoma metastasis has been documented for lymph node and pulmonary metastases. The C-C chemokine receptor type 7 (CCR7) promotes melanoma cell metastasis to the C-C motif ligand 21 (CCL21)-rich lymph nodes while the C-X-C chemokine receptor type 4 (CXCR4)/C-X-C motif chemokine ligand 12 (CXCL12) axis facilitates pulmonary metastasis [17,18]. Izraely et al. provided evidence for chemokine-mediated homing of disseminated melanoma cells to the brain using nude-BALB/c mice and implants of YDFR human melanoma cells to generate primary and brain metastatic tumors, from which cell lines were derived [19]. Although these cell lines were of the same genetic background, primary tumor-derived cells expressed low levels of the chemokine receptor CCR4, whereas brain-metastasizing melanoma cells expressed significantly higher levels of CCR4. CCR4 has already been shown to direct organ-specific metastasis of breast, colorectal, and gastric cancers [20,21,22]. Furthermore, “brain-derived soluble factors” upregulate CCR4 expression in melanoma cells and enhance the migration of brain-metastasizing melanoma cells specifically; however, whether these soluble factors are the brain-expressed CCR4 ligands, CCL17 or CCL22, and whether this signaling axis promotes melanoma brain metastasis remains to be determined [23]. Recently, an examination of the chemokine and cytokines (immunokines) within the cerebrospinal fluid of a small number of patients with melanoma brain metastases reported global reconfigurations of immunokine profiles. Lok et al. observed a significant correlation between poor clinical outcomes and alterations in several CCR4-binding immunokines, including CCL22, CCL4, and CCL17 [24]. These correlations suggest that changes in the intracranial “immunokine” profile may facilitate the establishment of brain metastases by melanoma cells through altered cytokine and receptor signaling that positively influences the retention or homing of disseminated melanoma cells to the brain. Whether these changes are a result of autocrine signaling by melanoma metastases or changes in the brain parenchyma in response to the metastases has not been determined. Further, it is not known whether such alterations enable metastasis progression or promote additional brain metastases. Additional studies like these need to be conducted with larger sample sizes to determine the significance of these differences and the correlation with disease progression and overall survival. Such data could then warrant additional studies to examine the effects of artificially modulating specific immunokines within the CSF to determine how these alterations influence melanoma brain metastasis.

Endothelin receptor B (EDNRB), a G protein-coupled receptor that is upregulated in melanoma metastases [25], is another molecule that could enable the preferential colonization of the brain by melanoma. Cruz-Munoz et al. used a xenograft mouse model of melanoma brain metastasis to demonstrate that EDNRB overexpression enhanced the metastatic potential of the implanted tumor cells, which resulted in more brain metastases compared with control tumor cells [26]. Blocking EDNRB specifically negated the enhanced metastatic phenotype and resulted in an increased overall survival in the mice. Furthermore, one of the EDNRB ligands, ET3, is highly expressed in the brain [27] and studies evaluating the clinical application of EDNRB antagonists are currently underway [18,28].

In the mid-1970s, researchers noted that melanoma cells had a very high density of receptors for, and strong affinity towards, neurotrophins such as nerve growth factor (NGF). This suggested that a ligand-receptor interaction could be the basis for melanoma’s predilection for the brain [29]. Subsequent studies have determined that p75^NTR^, a receptor for NGF, is highly upregulated in melanoma cells and associated with brain metastasis [30,31]. Other receptors such as TrkC, the putative receptor for neurotrophin-3 (NT-3), are also highly expressed on melanoma cells and suggest that neurotrophins may help recruit metastatic melanoma cells to the brain [32]. Interestingly, astrocytes found at the stromal—tumor interface of melanoma brain metastases display increased expression of neurotrophins like NGF and NT-3, further supporting the hypothesis that these ligands originate from the brain and support melanoma cell metastasis [33,34].

High endogenous expression of transforming growth factor-β2 (TGF-β2), which is an isoform of TGF-β [35], was able to direct melanoma cells to form brain metastases within the parenchyma of mice, while low expression of TGF-β2 induced brain metastasis within the ventricles and leptomeninges [36]. Reducing expression levels of TGF-β2 by knockdown with shRNAs significantly inhibited brain parenchyma metastasis, suggesting that melanoma cell expression of TGF-β2 controls, or is required for, the site-specific colonization of metastatic melanoma. Recently, the levels of a microRNA (miRNA) that controls the expression of TGF-β2 was shown to be dramatically decreased, with a concomitant increase in expression of TGF-β2, in human melanoma cell lines [37]. Forced expression of miR-328 inhibited proliferation by inducing cell cycle arrest in human melanoma cells [37]. Future studies should determine how miR-328 levels are controlled and whether it functions through TGF-β2 to regulate site-specific melanoma brain metastasis formation. In addition, it will be interesting to understand how TGF-β2 determines the fate of melanoma cell localization intracranially. These studies suggest that both melanoma cell autonomous and non-cell-autonomous mechanisms are responsible for the chemokine- and receptor-mediated recruitment, homing, and retention of melanoma cells to the intracranial vasculature. Once localized within the brain, melanoma cells receive tissue-derived cues that potentiate extravasation, the transmigration of metastasizing cells out of the vasculature and across the BBB.

### 2.2. Extravasation

The BBB presents a formidable border that tumor cells must cross in order to establish residence within the brain. The BBB functions as a highly selective and tight interface between the blood within the microvasculature and the parenchyma tissue of the brain. It is composed of continuous, non-fenestrated endothelial cells that are reinforced by an underlying basement membrane and connected by tight junctions [38]. Inter-endothelial tight junctions, composed of transmembrane complexes that link adjacent cells, are extremely tight and thus prevent the passive diffusion of ions and other polar solutes between endothelial cells [39]. Cerebral endothelial cells are supported on their extravascular side, next to the collagen-rich extracellular matrix, by a tightly-associated layer of pericytes along with the foot processes of astrocytes that form a perivascular sheath [38]. One of the main functions of the BBB is to strictly regulate the flow of ions, nutrients, and cells into the brain parenchyma. How melanoma cells are able to cross the BBB is still not completely understood, as it is difficult to study these processes in vivo or in vitro. Kienast et al. noted that successful transendothelial migration of metastasizing cells was highly inefficient and occurred slowly, taking up to 14 days [9]. Several mechanisms for how the cerebral endothelium and arrested melanoma cells interact to enable extravasation, which is thought to occur via a paracellular route, have been described.

Heparanase (HPSE), an enzyme that degrades heparan sulfate chains of proteoglycans enriched in endothelial cell layers, is upregulated in many cancers and correlates with metastatic potential [40]. HPSE may act in a brain-specific manner allowing penetration of the BBB by metastatic melanoma cells [41,42,43]. The presence of HPSE increases invasion of melanoma cells into brain tissues in brain slice models [43]. Furthermore, co-incubation of brain metastatic melanoma cells with astrocytes results in elevated HPSE activity and an increased invasive phenotype in vitro [42]. The role of HPSE in brain-specific metastasis is further supported by the fact that brain-derived neurotrophins, such as NGF, enhance HPSE activity [44]. More recently, miRNA suppression of HPSE has been shown to inhibit melanoma migration, invasion, and adhesion, but examination of melanoma brain metastasis formation with knockdown of HPSE was not reported [45]. miRNA-mediated knockdown of HPSE does suppress breast cancer brain metastasis [46]. Whether targeting HPSE is a therapeutic option for inhibiting melanoma brain metastasis remains to be determined [41].

In vitro studies utilizing confluent brain endothelial cells as a model of the BBB examined transmigration of fluorescently labeled human A2058 and murine B16/F10 melanoma cells lines [47]. Transmigration occurred via the paracellular transmigration pathway, in a process that caused the disruption of the tight junction proteins occludin and claudin-5. Transmigration was partially dependent upon the gelatinolytic serine protease, seprase, as either Pefabloc, a serine protease inhibitor, or siRNA-mediated knockdown of seprase, decreased transmigration through interendothelial junctions [47]. An additional report utilizing an in vitro brain endothelial BBB model demonstrated that melanoma cells transmigrate more rapidly, and have an increased ability to impair tight junctions, when compared withbreast cancer cells [13]. Molnár et al. also showed that adhesion of melanoma cells to the brain endothelial cells requires Rac or PI3K activity, as inhibition of either impaired melanoma cell adhesion and the early phase of transmigration [13]. These studies utilized in vitro models of the BBB to dissect the mechanisms of melanoma cell extravasation, which have proven to be informative. It will be interesting to determine whether these processes are required for the in vivo establishment of melanoma brain metastases.

Several reports have implicated a role for Connexin-26 (Cx26) or Connexin-43 (Cx43), which are the only gap junction proteins expressed in melanocytes [48,49], as having a role in melanoma brain metastasis. Stoletov et al. demonstrated that melanoma cells utilize the gap junction protein Cx26 to associate with the cerebral endothelial cells in chickens during the initiation of brain metastasis [50]. Silencing of Cx26 in melanoma cells inhibited extravasation and in vivo brain colonization [50]. However, these studies were performed with only one mouse cell line (B16) that expresses Cx26 but not Cx43. Other reports show that patient samples of malignant melanomas have high expression of Cx43 while Cx26 expression is nearly absent [51]. More recently, Cx43 overexpression was found to inhibit melanoma proliferation and metastasis by inducing TGF-α-mediated apoptosis but brain metastasis was not reported [52,53]. Thus, additional studies on the role of gap junctions in melanoma extravasation into the brain parenchyma are needed.

Recently, Herwig et al. demonstrated a role for high levels of extracellular S100A4, a member of the S100 calcium-binding protein family, in decreasing inter-endothelial tight junction integrity by using A375 human melanoma cells and an in vitro BBB model [54,55]. Paracrine-mediated signaling of S100A4 secreted from melanoma cells with its cognate receptor, receptor for advanced glycation end-products (RAGE), on endothelial cells enabled transmigration. This occurred by reducing expression of the interendothelial tight junction molecules, occludin and VE-cadherin, and inducing a loss of endothelial tightness, as determined by decreases in transendothelial electrical resistance (TEER). They also revealed that brain metastasis formation by A375 cells injected intracardially into athymic mice was increased with overexpression of S100A4/RAGE [54,55]. S100A4/RAGE signaling has been linked to the pro-migratory, invasive, and metastatic characteristics of several tumor types, including thyroid, prostate, and colorectal [56,57,58]. S100A4/RAGE signals through diaphanous-1 (thyroid cancer), nuclear Factor κ B (NFĸB) (prostate cancer), and/or mitogen-activated protein kinase (MAPK)/extracellular regulated kinase (ERK) (colorectal) pathways and it will be informative to investigate which intracellular pathways are modulated by S100A4/RAGE paracrine signaling to promote melanoma transmigration. S100A4 also stimulates the production and secretion of MMP-9, which likely aids in degrading the extracellular matrix to promote transmigration [59]. A neutralizing monoclonal antibody targeting S100A4, 5C3, has been shown to prevent endothelial cell migration of melanoma cells in an immunodeficient mouse xenograft model [59], thus additional research on the clinically relevant efficacy of targeting S100A4 should be explored.

Pleckstrin homology domain-containing family A member 5 (PLEKHA5) is a recently described candidate to emerge from integrated comparisons of clinical melanoma samples and cell lines with a “brain homing” phenotype [60]. High PLEKHA5 expression in melanoma patients was associated with decreased brain metastasis-free survival, which is defined as the elapsed time from diagnosis of first distant metastasis to the diagnosis of brain metastasis. PLEKHA5 silencing decreased melanoma cell survival and inhibited transmigration through an in vitro BBB model, suggesting that PLEKHA5 plays a role in viability and extravasation into the brain parenchyma. It will be interesting to determine the mechanism by which PLEKHA5 mediates intracranial extravasation and whether it is important for melanoma brain metastasis formation in vivo.

Recently, bidirectional astrocyte-melanoma signaling has been shown to reciprocally stimulate an increase in the invasiveness of melanoma cells. Brain-metastasizing melanoma cells stimulate astrocytes to express the pro-inflammatory IL-23 cytokine resulting in IL-23-mediated stimulation of melanoma cells to secrete matrix metalloproteinase 2 (MMP2) [61]. MMP2 production increases extravasation by degrading collagen type IV, a major component of the extracellular matrix (ECM) surrounding the brain endothelial cells [61]. Increased MMP2 secretion via IL-23 signaling may be mediated through signal transducer and activator of transcription 3 (STAT3) [62] as STAT3 activity regulates MMP2 expression and human brain metastatic melanoma cells and tissue biopsies show increased STAT3 activity as compared to cutaneous melanoma cells and extracranial lesions [63]. Up-regulation of MMP2 has been previously tied to melanoma invasion as it was shown to be upregulated in highly metastatic human melanoma cell lines and strong MMP2 expression significantly correlates with advanced metastatic disease and worse overall survival [64,65]. In vitro BBB models have demonstrated that MMP2 expression influences transmigration of melanoma cells across human brain endothelial cells and additionally supports a role for other MMPs (e.g., MMP7, 10, 11, 13) in potentially mediating degradation of the extracellular matrix to facilitate extravasation [66].

TGF-β2, discussed above in regulating melanoma brain tropism, also controls the permeability of endothelial cell monolayers, and may thus influence melanoma cell extravasation as well. TGF-β2 was shown to decrease endothelial barrier tightness in in vitro BBB assays by regulating MMP secretion and down-regulating endothelial tight junction proteins [67]. Studies to determine whether TGF-β2 functions similarly to support and enable transmigration of melanoma cells across the BBB in vivo have not been reported.

These mechanisms highlight the incredibly complex nature of melanoma cell interactions with the brain endothelium. Additional studies detailing how brain-tropic melanoma cells transmigrate across the endothelial BBB are underway, aided by the development of more refined in vitro BBB models [68], and will likely reveal diverse and complex mechanisms that may enable additional targeting of the process of extravasation during the establishment of brain metastases.

### 2.3. Perivascular Positioning and Growth by Vessel Co-Option

The successful establishment of transmigrated melanoma cells as metastases is dependent upon their ability to secure necessary access to the microvasculature by maintaining perivascular localization and stimulating angiogenesis. Transmigrated melanoma cells gain access to the blood supply in two ways—persistent close contact to microvessels and perivascular growth by vessel co-option [9]. Kienast et al. noted that melanoma cells that had extravasated, but lacked intimate association with the abluminal side of the microvasculature, failed to proliferate and thus regressed [9]. Evidence for the high degree of proficiency that melanoma cells exhibit for vascular co-option has been observed in studies detailing the histomorphological characteristics of brain metastases in humans. When compared with lung, breast, colorectal, and kidney cancers, vascular co-option is most commonly observed in melanoma metastases [69]. Unfortunately, the mechanisms whereby brain metastastic melanoma cells secure necessary metabolites through vascular co-option and angiogenesis are still poorly understood. However, many of the aforementioned molecules involved in extravasation have also been shown to play roles in concomitantly fueling vessel co-option. For instance, Cx26 is also implicated in vessel co-option during melanoma metastasis to the brain [50]. STAT3 activation promotes brain metastasis by stimulating vascular remodeling through increased expression of basic fibroblast growth factor (bFGF), vascular endothelial growth factor (VEGF), and MMP-2 [63,70]. S100A4 overexpression in M21 melanoma cells simulates tumor vascularization in human melanoma xenograft models in addition to inducing transmigration [59]. Secretion of S100A4 was shown to synergize with VEGF to strongly promote angiogenesis and enable tumor growth [59]. Kienast et al. noted that the human melanoma cell line they injected intracardially, MDA-MB-435, lacked VEGF-A expression, suggesting that other mechanisms of vessel co-option and angiogenesis may be present and utilized by melanoma cells. A recent analysis of angiogenesis-related gene expression in human brain melanoma metastases documented a more than 50-fold increase in C-X-C motif chemokine ligand 10 (CXCL10), carcinoembryonic antigen related cell adhesion molecule 1 (CEACAM1), platelet and endothelial cell adhesion molecule 1 (PECAM1), cluster of differentiation 117 (CD117/KIT), collagen type IV alpha 2 (COL4A2), collagen type I alpha 1 (COL1A1), and heparan sulfate proteoglycan 2 (HSPG2) [71]. However, to what extent these genes contribute to neovascularization in melanoma brain metastases formation will require additional studies. Interestingly, the phosphatidylinositol-4,5-bisphosphate 3-kinase (PI3K)-protein kinase B (PKB/AKT) pathway, discussed in more detail in the following section, impacts vascular co-option and angiogenesis via its role in regulating VEGF, hypoxia-inducible factor 1-alpha (HIF1α), and angiopoietin-2 (ANG2) [72,73,74,75].

## 3. AKT Signaling and Melanoma Brain Metastasis

Aberrations that lead to over-activation of the PI3K/AKT signaling pathway are some of the most common events in human cancer [76]. In melanoma, oncogenic alterations of this pathway are observed in up to 70% of patient tumors and are associated with disease progression [77,78,79]. The PI3K/AKT pathway is an important regulator of cell cycle progression and therefore is a frequent contributor to cellular transformation when normal function is compromised via genetic or epigenetic modifications [80]. Conventional activation of the pathway is initiated at the cell surface by the phosphorylation of receptor tyrosine kinases (RTKs) in response to mitogen stimulation. This event induces the recruitment of phosphoinositide-3-kinase regulatory subunit 1 (PIK3R1) and phosphatidylinositol 3-kinase (PI3KCA), which form a heterodimer to produce PI3K at the plasma membrane. Here, PI3K phosphorylates phosphatidylionositol-4,5-bisphosphate (PIP2) to phosphatidylinositol-3,4,5,triphosphate (PIP3), a phospholipid that attracts and binds the serine-threonine-specific kinase AKT (Protein Kinase B). A subsequent conformational change in AKT permits its phosphorylation and activation by 3-phosphoinositide-dependent protein kinase 1 (PDK1) and mammalian target of rapamycin complex 2 (mTORC2) [81].

AKT is a major signaling hub that executes a myriad of biological responses including transcription, glucose metabolism, cell migration, cell proliferation, apoptosis, and angiogenesis [82]. Various mechanisms of PI3K/AKT hyperactivation have been described in the melanoma literature, including RTK amplification, activating mutations in and increased gene expression of PI3KCA, as well as genomic amplification and activating mutations in AKT [76,83,84,85]. However, the most common mechanism by which the PI3K/AKT pathway acquires hyperactivity is via inactivation or deletion of phosphatase and tensin homolog deleted on chromosome 10 (PTEN). PTEN is a lipid phosphatase that antagonizes the function of PI3K through dephosphorylation of PIP3; the conversion of PIP3 to PIP2 diminishes recruitment of AKT to the plasma membrane and suppresses AKT phosphorylation [86]. Ten to thirty percent of melanomas are subject to loss, mutation, or epigenetic silencing of PTEN [87], which results in increases in the level of phospho-AKT (P-AKT).

Although evidence that the PI3K/AKT pathway plays a role in melanoma initiation has been demonstrated in functional experiments (reviewed in [88]), studies comparing the levels of PTEN in primary versus metastatic melanomas suggest that PTEN inactivation, the predominant driver of aberrant PI3K/AKT activity in melanoma, is a late event [89,90]. Indeed, the transformation of normal nevi to melanoma in situ, and from melanoma in situ to metastasis, is marked by a progressive accumulation of P-AKT. Primary melanomas with robust P-AKT expression also correlate with a worse prognosis [77,91]. Furthermore, Govindarajan et al. discovered that over-expression of AKT in a radially growing melanoma cell line conferred an invasive, vertical growth phenotype when implanted in mice [92]. These studies implicate PI3K/AKT over-activation as a facilitator of melanoma progression and metastasis.

As advances in our understanding of PI3K/AKT hyperactivity in melanoma progression and metastasis continue to be made, many studies have shifted focus to brain tropism, the results of which are reshaping the way we think about the PI3K/AKT pathway and melanoma brain metastasis. Several studies that emphasize the temporal and spatial dynamics of PI3K/AKT activation during melanoma metastasis have highlighted the importance of PI3K/AKT hyperactivity in metastasis to the brain. An integrated analysis of patients with BRAF^V600^- or NRAS-mutated, and PTEN loss stage IIIB/C melanomas, found that PTEN loss corresponded to a shorter time to brain metastasis and reduced overall survival in BRAF^V600^-mutated melanomas [93]. Immunohistochemical analyses of patient-matched brain and extracranial melanoma metastases by Niessner et al. also showed that PTEN was downregulated and P-AKT was upregulated in brain metastases, whereas the opposite effect was true in extracranial metastases [94]. A similar study revealed that although P-AKT levels between patient-matched brain, lung, or liver metastases and their corresponding regional metastases did not differ, there was a significant difference in P-AKT levels between distant metastatic sites, with the highest P-AKT levels residing in brain metastases [78]. A follow-up study using DNA, RNA, and protein-based analyses designed to better understand the molecular pathogenesis of melanoma brain metastases found that brain metastases exhibited specific molecular patterns distinct from extracranial metastases. Specifically, brain metastases displayed a PI3K/AKT activation signature as assessed by P-AKT levels and activation of glycogen synthase kinase-3 α/β (GSK3α/β) and proline-rich Akt substrate of 40 kDa (PRAS40), downstream effectors of AKT. However, results of these analyses did not yield a conclusive rationale to account for these observations as no copy number gains or mutations of PIK3CA or AKT were found in brain metastasis-only tissue from matched samples. Fewer copies of PTEN were noted in 20% of brain metastases compared with extracranial metastases, nevertheless, neither reverse phase protein array (RPPA) or IHC revealed any decrease in PTEN expression between matched samples [95]. These results highlight the complex relationship between PTEN loss and AKT activation.

One recent in vivo study demonstrated that AKT activation is an important contributor to melanoma lung and brain metastasis. Using an autochthonous model of melanoma, Cho et al. demonstrated an important role for AKT1 activation on the incidence of brain metastasis in the presence or absence of Pten. In vivo experiments showed that when oncogenic alterations of BRAF^V600E^, Cdkn2a^−/−^, and Pten^−/−^ were induced in the melanocytes of immunocompetent mice, melanomas were generated in 100% of animals but fewer than 10% of the animals developed metastases. However, when a constitutively active form of AKT1 was expressed in this context nearly 80% of the animals developed brain metastases. Furthermore, RPPA analyses demonstrated differential gene expression and activation signatures in mTOR signaling pathway components between tumor samples with activated AKT1 versus Pten loss [96]. These findings indicate that the downstream activation profile of AKT1 does not mirror that of Pten loss. It is possible that the stimulation of different AKT isoforms may yield distinct cellular responses. The consequence of AKT2 or AKT3 (the predominantly active isoform in melanoma) [91] activation in this context is currently under investigation.

The PI3K/AKT pathway is linked to several later steps of melanoma metastasis and has been shown to regulate cell adhesion, extravasation, degradation of extracellular matrix proteins, and angiogenesis. These mechanisms include signaling between the PI3K/AKT pathway and CCR4, HSPE (heparanase), VEGF, STAT3, or Cx43. Molnár et al. recently showed that adhesion of melanoma cells to the brain endothelial cells requires PI3K activity, as its inhibition impaired melanoma cell adhesion and the early phase of transmigration [13]. CCL22, a ligand for CCR4, was found to increase P-AKT in primary-derived cells but decrease P-AKT in brain-derived cells, although the biological rationale behind these antagonistic responses or how the responses are orchestrated are not clear [19]. The increased invasiveness of melanoma cells into the brain tissues of brain slice models through the action of HSPE is at least partially attributed to the ability of HSPE to stimulate the PI3K/AKT pathway [43,97]. Inhibition of PI3K reduces melanoma cell transmigration through human umbilical vein endothelial cells (HUVECs), suggesting that this process is mediated through the PI3K/AKT signaling hub [73]. AKT also phosphorylates Ser369 and Ser373 on Cx43 [98], and while the consequence of this event in the context of melanoma biology remains to be determined, Cx43 promotes melanoma brain colonization through enhancement of cell extravasation and co-option of blood vessels [50]. Similar to Cx43, the cytokine and growth factor-dependent transcription factor, STAT3, has shown interconnectivity to the PI3K/AKT pathway in transformed murine cells, although many details of this complex interactive network have yet to be elucidated [99].

The microenvironment also appears to influence the gene expression patterns of melanoma cells present in the brain. In an in vitro study, human metastatic melanoma cells were assessed for PI3K/AKT activity and cell invasion after exposure to conditions that simulate the brain microenvironment. Patient-matched brain and extracranial metastases were immersed in astrocyte- or fibroblast-conditioned medium. Cells exposed to astrocyte-conditioned medium responded by elevating levels of P-AKT and increasing invasiveness; this was not observed for cells exposed to fibroblast-conditioned medium [94]. In an impressive illustration of the power of the brain tumor microenvironment to influence the transcriptome of cancer cells, Park et al. used competitive cross-species hybridization of microarray experiments to highlight the ability of the brain microenvironment to dictate the transcriptional profiles and phenotypes of cancer cells. In this study, breast, colon, lung, or melanoma cells were transplanted into the brains or skin of immunodeficient mice. Tumors were later excised and the mRNA signatures of the human cancer cells analyzed. While 4213 genes significantly differed among melanomas transplanted subcutaneously, reflective of the intrinsic genetic differences between cancer types, only 21 genes differed among tumors transplanted into the brain. These experiments highlight the ability of the brain microenvironment to override cell-specific transcriptomes and execute radical reprogramming of invading cells [100].

A recently discovered, non-cell-autonomous mechanism by which the brain microenvironment stimulates the PI3K/AKT pathway is through astrocyte-derived exosomes that deliver *PTEN*-targeting miRNAs to melanoma cells. Zhang et al. discovered that *PTEN*-targeting miRNAs, previously determined to be encoded by the miR-17-92 allele [101,102,103,104], are excreted from brain astrocytes in exosomes, thereby mediating an intercellular silencing of PTEN and concomitant activation of PI3K/AKT in metastatic melanoma cells. Silencing of miR-17-92 in astrocytes, or inhibiting exosome secretion, rescued PTEN loss in melanoma cells and suppressed brain metastasis [105]. These experiments demonstrate how the brain milieu generates temporal and reversible epigenetic alterations in melanoma cells, thereby influencing their metastatic potential.

Substantial evidence exists that over-activation of the PI3K/AKT pathway is a causal factor in melanoma brain metastasis through both tumor cell autonomous and non-cell-autonomous mechanisms. Furthermore, alterations occur in many different members of this pathway in multiple cancer types [76]. The studies discussed here emphasize the numerous channels of interconnectivity to other signaling pathways, and demonstrate that we lack a complete understanding of how these modifications translate to different but widespread molecular changes that promote melanoma brain metastasis. The elucidation of this network will allow the identification of key pathway nodes that represent potential therapeutic targets to disrupt the process of melanoma brain metastasis.

## 4. Animal Models of Melanoma Brain Metastasis

**Figure 2:**
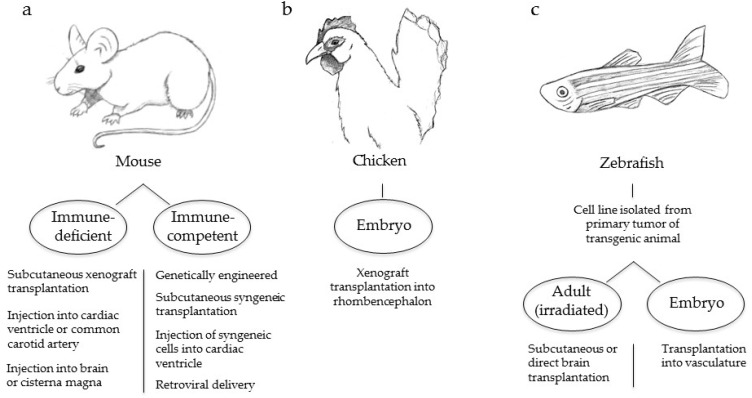
Animal models used to investigate melanoma brain metastasis. (**a**) Immunodeficient and immunocompetent mouse models; (**b**) Chicken embryo model; (**c**) Zebrafish adult and embryo models.

There is a strong need to develop experimental models that accurately mimic the human disease both at the molecular and cellular levels such that more effective therapeutic strategies to prevent, delay, and treat melanoma brain metastases can be developed. Melanoma brain metastases have been successfully generated following transplantation of melanoma cells into immunodeficient mice (Figure 2a) [70,106,107,108]. Studies that introduce melanoma cells subcutaneously have the advantage of being able to recreate and analyze the full range of metastatic events necessary for the development of brain metastases from the primary tumor [19,106,109]. However, these models often have a long latency and require excision of the primary tumor followed by serial passage of the tumor cells in vivo to derive cell lines that reproducibly generate brain metastases.

Another method used to study melanoma brain metastasis is through injection of melanoma cells via the left cardiac ventricle or common carotid artery [36,63,70,108,109,110]. The direct introduction of cells into the circulation reduces disease latency and produces a high incidence of brain metastases. A disadvantage of this method is that early steps in the metastatic cascade are bypassed, and therefore this method is primarily useful for studying BBB extravasation and brain colonization. Direct injection of melanoma cells into the brain or the cisterna magna of immunodeficient mice has also been successfully employed to generate viable tumors that disperse and proliferate in the brain [107,111]. However, while this method is useful for assessing brain colonization and angiogenesis, it does not allow full analysis of the multi-step process of metastasis.

In immune-competent models, melanoma cells must evade the immune system during disease progression and therefore these models more accurately mimic the human disease. In the metallothionein (MT)/*ret* transgenic model, the human *ret* transgene is driven by the mouse MT promoter-enhancer, which activates MAPK signaling and promotes the development of spontaneous melanomas; a subset of these mice develop metastases of the brain and other distal sites [112,113]. The *ret* transgene system has subsequently been used to develop a spontaneous model of melanoma brain metastasis through the use of transplantable cells. Schwartz et al. utilized a melanoma cell line derived from *ret*-generated tumors and performed subcutaneous injections into syngeneic C57BL/6 mice to grow primary tumors that eventually metastasized to the brain in nearly 75% of mice [112,114]. However, alterations in RET have not been reported to occur in human melanomas [112].

The introduction of syngeneic murine melanoma cells via intracardiac injection in an immunocompetent model also has been performed and shown to produce brain metastases. In a study designed primarily to characterize brain metastasis growth patterns using magnetic resonance imaging, Morsi et al. injected murine B16F10 melanoma cells into the left cardiac ventricle of C57BL/6 mice. Contrary to the intracarotid artery method of injection, left ventricle injections allow cells to circulate freely within the vascular system, an approach that permits cells indiscriminate access to all major organs. This is a useful technique to determine the proclivity of a specific cell line to favor particular sites of metastasis. Brain metastases in this experiment were produced in approximately one quarter of the mice and with rapid onset [115]. However, as with left ventricle injections in immunodeficient mice, early steps in metastasis are bypassed. This study also demonstrated a propensity of B16F10 melanoma cells to seed and proliferate other extracranial sites.

Recently, an immune-competent autochthonous model of melanoma brain metastasis was developed based on the avian retroviral replication-competent ALV LTR splice acceptor (RCAS)/tumor virus A (TVA) system, whereby ectopic expression of the avian TVA receptor is expressed in melanocytes under control of the dopachrome tautomerase (DCT) promoter. Avian cells that produce an RCAS virus carrying an oncogene of interest are subcutaneously injected into DCT-TVA mice. The virus targets melanocytes and the delivered oncogene is permanently integrated into host DNA for long-term ectopic gene expression. This model also utilizes floxed alleles in melanoma-relevant genes to express BRAF^V600E^, inactivate the *Cdkn2a* locus, and inactivate *Pten*, via delivery of a second virus that carries Cre recombinase. Cho et al. used this model to generate melanoma in 100% of mice and brain metastases in 79% of mice through expression of a constitutively active form of AKT1 in this context [96]. This system offers a reproducible method whereby primary tumors and brain metastases are produced quickly and reliably using specific combinations of oncogenic alterations commonly found in human melanoma. Other oncogenes of interest can also be quickly tested for their roles in melanoma brain metastasis based on physiologically relevant genetic mutations that occur in the human disease.

Non-murine preclinical animal models provide additional options to study melanoma brain metastasis. Because malignant transformation displays some parallels to the physiology of embryonic cells, Busch et al. set out to explore the effects of an embryonic-rich environment on the invasiveness of human melanoma cells in the brain. To accomplish this objective, the chicken embryo was chosen as a model in which to perform xenotransplantation experiments (Figure 2b). Melanoma cells injected into the rhombencephalon of embryos were found to reproduce the invasive characteristics of the human disease. This model offers the potential of studying the disease spread of multiple human melanoma cell lines. Embryos are easily accessible and tumor spread is rapid. However, the clinical relevance of malignant cell behavior in an environment saturated by growth-promoting undifferentiated cells remains unclear. Furthermore, while this method is useful for assessing brain colonization and angiogenesis, it does not allow full analysis of the multi-step process of metastasis.

In recent years the zebrafish has emerged as a useful animal model to study cancer due in part to the transparency of its embryos and the ability to map the fate of single cells with unprecedented detail through high-resolution imaging. For this reason, this model is an attractive candidate to assess the spatio-temporal dynamics of disease spread. In order to perform a comprehensive quantitative analysis of metastatic biology, Heilmann et al. used a transgenic *capser* zebrafish model with the genotype *BRAF^V600E^; p53^−/−^; mitfa^−/−^*, a strain devoid of melanocytes. Fish were injected with a plasmid that uses separate mitfa promoters to drive expression of microphthalmia-associated transcription factor (MITF) and enhanced green fluorescence protein (EGFP). Upon in vivo rescue of MITF expression, patches of EGFP-positive melanocytes formed, a subset of which underwent transformation. Stable cell lines were generated from primary tumors, one of which (ZMEL1) was transplanted subcutaneously into irradiated adult *casper* fish, or the vasculature of *casper* embryos (Figure 2c). In both circumstances, primary tumors and subsequent metastases to multiple sites including the head were observed within two weeks [116]. Advantages of this model include the rapid generation of progeny, the ability to track the fate of single metastatic cells that quickly spread, and the potential to use gene editing of cell lines to perform genome-wide in vivo screens to more clearly define the contribution of a specific gene in metastasis. However, similar to the chicken embryo model, experiments in the growth-promoting environment of the fish embryo lack physiological relevancy. Likewise, these experiments require that adult fish are irradiated to compromise adaptive immunity prior to the introduction of melanoma cells.

Current animal models of melanoma brain metastasis have made advances in our understanding of the melanoma cerebrotropism but many questions remained unanswered. Improvements in present models and the development of new models are needed to increase our understanding of the biology of melanoma brain metastasis. This will require sophisticated models that minimize the fundamental limitations imposed by differences in species biology, and maximize the ability to mimic the heterogeneity of the human disease in a relevant microenvironment. Goals will be to identify useful biomarkers of brain metastasis, interrogate and accurately describe molecular mechanisms of these processes, assess the efficacy of existing and experimental therapeutics, as well as develop new effective treatment strategies. In vivo models capable of identifying how and why brain metastasis occurs will provide a foundation upon which important new scientific breakthroughs and treatment strategies can be translated to the clinic for improved patient care.

## 5. Melanoma Brain Metastasis Therapies

Brain metastases are a major complication of metastatic melanoma and are responsible for up to half of all melanoma deaths [117,118,119,120]. Among all cancers that frequently metastasize to the brain, including breast, lung, colon, and renal, melanomas have the highest frequency for colonizing this organ [121,122,123,124]. Between 6% and 43% of melanoma patients present with brain metastases at stage IV diagnosis and nearly 75% of autopsy reports identify CNS involvement [120,125,126], as tumor burden in terminal patients is often higher than clinically realized. Brain metastasis bodes very unfavorably for prognosis in melanoma and overall survival time for patients with intracranial metastases ranges between 4 and 9 months after diagnosis [118,126,127]. A graded prognostic assessment (GPA) was introduced by Sperduto et al. to systematically and more accurately determine the prognosis of patients with brain metastases. The diagnosis-specific GPA considers age, Karnofsky performance score (KPS: a measure of the ability of a patient to perform ordinary tasks), extracranial metastases, and the number of brain metastases [128]. A melanoma-specific GPA was revised recently and prognosis was determined to be based primarily on KPS and the number of brain metastases, with a low KPS score and more total brain metastases indicative of a poor prognosis (median OS of 3.4 months) and a higher KPS with fewer metastases showing a better prognosis (median OS of 13.2 months) [129]. However, with the advance of targeted therapies such as BRAF and MEK inhibitors and immune checkpoint inhibitors (discussed in detail below), the utility and accuracy of the Sperduto GPA for determining the prognosis of patients with melanoma brain metastases will be altered and thus require additional revisions to remain a useful prognostic tool [130].

The morbidity and mortality associated with melanoma brain metastases are most often attributed to hemorrhage and increased intracranial pressure. Among all brain malignancies, melanoma brain metastases have the highest risk of hemorrhage, with 27%–40% of all intracranial lesions showing active hemorrhage on neuroimaging and up to 71% of patients with melanoma brain metastases showing evidence of prior hemorrhage by histopathology [131,132]. In addition to hemorrhage, brain metastases are associated with other complications such as hydrocephalus from obstructed flow of cerebrospinal fluid and local mass effect by tumor expansion [124]. These complications place the patient at risk for increased intracranial pressure and commonly manifest as headaches, nausea, mental status change, vomiting, cranial nerve palsies, visual deficits, hemiparesis, and sensory loss [124,131]. Focal and generalized seizures are also common sequelae of brain metastases and further add to the morbidity of this complication.

Current clinical therapy for melanoma brain metastasis can be divided into three broad categories: palliative, definitive, and investigational [124,133]. Palliative care includes steroids to reduce inflammation caused by hemorrhage and edema, anticonvulsants to combat seizures secondary to the metastatic lesion, and anticoagulants to prevent post-operative thromboembolic disease. Definitive therapy includes radiation, either whole brain radiation therapy or stereotactic radiosurgery (SRS), surgical resection, and chemotherapy [127,134]. Investigational therapies encompass the recently approved targeted and immune therapies, which are beginning to show great promise [135,136,137].

General recommendations for a particular therapy, which take into account the estimated prognosis of the patient and overall aim of treatment, are based on several criteria, including the size of the brain metastasis, the location of the brain metastasis, and the presence of other brain metastases [124,136,138,139]. Other important factors include age, the presence and/or extent of extra-cranial metastases, KPS, and overall health of the patient [136]. The median overall survival of brain metastasis patients opting out of definitive therapy is approximately 1–2 months [120,140].

### 5.1. Surgery

Surgical resection is an option if the metastasis is solitary or limited in number and located in a surgically accessible area [141]. Surgery may be recommended if the metastasis is symptomatic and/or requires a diagnosis but biopsy of an alternative area is not feasible [142,143,144]. Immediate tumor de-bulking, definitive targeting of the lesion, and histological and molecular characterization of resected tissues, while alleviating symptomatic mass effects of the tumor, are all advantages of surgical resection. Melanoma brain metastasis patients who are candidates for surgical resection and initially receive this treatment have a median overall survival of 9.83 months [118,145]. Patients with multiple melanoma brain metastases or disseminated carcinomatous cell spreading in the brain also known as “miliary metastases” are not surgical candidates and are recommended alternative therapies such as radiation. For patients who undergo surgical resection, post-operative radiation therapy in the form of SRS or whole brain radiation therapy (WBRT) is common [141,146,147].

### 5.2. Stereotactic Radiosurgery

Melanoma is notoriously radioresistant; however, radiation therapy is an integral part of the standard of care for patients with brain metastasis [144]. SRS is a targeted approach that involves the administration of a single fraction of ionizing radiation via several converging beams onto a targeted site. The delivery of high-dose, focally targeted radiation to a confined area has been demonstrated to minimize extraneous exposure and mitigate the undesired side-effects of radiotherapy [148,149]. Evidence-based guidelines from the American Society for Radiation Oncology recommend that SRS, rather than WBRT, be utilized to treat patients with limited brain disease [144]. SRS is most effective when treating patients who have small lesions numbering fewer than 3 [143] and confers a survival advantage for patients who are ≤50 years of age [150]. Melanoma patients recommended to receive SRS and initially treated with this modality have a median overall survival of 7.69 months [118]. This OS for SRS has been corroborated recently (8.1 months) in a report that also devised novel risk scores for OS and intracranial failure [151]. Chowdhury et al. report a model to determine risk scores of OS based on performance status, extracranial disease status, number of lesions, and gender, thereby providing additional predictors for prognosis and treatment strategy [151]. Treatment of brain metastases with SRS will also benefit from considering and incorporating individual histologies into treatment plans, especially when used in combination with systemic therapies, as such studies will be instrumental in determining outcomes [144].

### 5.3. Whole-Brain Radiation Therapy

WBRT is untargeted over the entire brain and therefore has the potential to cause acute adverse effects, including cerebellar dysfunction and cognitive deterioration, which vary by duration and severity [152]. Despite the radioresistant nature of melanoma, WBRT is used when the number of brain metastases is such that it precludes a more targeted approach [140]. WBRT has been shown to prevent intracranial relapse, but has little impact on overall survival [149]. A prospective study by Aoyama et al. demonstrated that the use of WBRT with SRS did not improve the survival for patients with 1–4 intracranial lesions, as compared with SRS alone [149]. Patients receiving WBRT have a median survival of 3.86 months [118,153]. A recent study that systematically reviewed the outcomes reported in 73 articles published between 1995 and 2014 also showed that WBRT has no significant impact on overall survival, but that combining it with SRS improved the brain relapse rate [153]. Because of the promise that targeted or immune-based therapies have shown (discussed below), combining them with SRS may eliminate the need for WBRT [153].

### 5.4. Chemotherapy

Standard chemotherapy for metastatic melanoma in the brain has not proven effective. Temozolamide, fotemustine, and thalidomides are used clinically in combination or alone but have very low response rates ranging between 7% and 10% and patients receiving chemotherapy alone to treat melanoma brain metastases have a median overall survival of 4.64 months [118,153,154,155,156]. Presumably this is due to the low intracranial activity of chemotherapeutics based on their inability to bypass the BBB.

### 5.5. Targeted Therapies

Targeted therapies include small molecules that inhibit the hyper-activated MAPK signaling pathway or antibodies that enhance the immune system response, but these are not currently standard of care for treatment of melanoma brain metastasis, largely because of the presupposed difficulties of bypassing the BBB. Until recently, melanoma patients with active brain metastases have been excluded from clinical trials that test their efficacy. However, several new studies have demonstrated that they are at least partially efficacious for treating intracranial lesions and are thus being considered for further investigation [135,137]. A systematic review of current clinical trials identified survival outcomes of melanoma brain metastasis patients who were treated with MAPK inhibitors and/or immune-based checkpoint blockade and reported that both have evidence of clinical activity and may increase OS in these patients [137].

### 5.6. MAPK Pathway Inhibitors

Approximately 40%–50% of melanoma patients have activating mutations in BRAF at valine 600 (90% are BRAF^V600E^) and another 15%–25% have mutations in the neuroblastoma RAS viral oncogene homolog (NRAS), and either alteration results in constitutive activation of the MAPK pathway [156]. Thus, inhibitors of the MAPK pathway are therapeutically relevant to a significant number of patients with metastatic melanoma. Inhibitors of mutant BRAF^V600^ have garnered much attention based on their impressive efficacy in treating metastatic melanoma, as rapid, systemic responses are frequently seen within weeks of the onset of treatment. These findings have sparked new studies designed to address the efficacy of BRAF^V600^ inhibitors against intracranial lesions. Two BRAF^V600^ inhibitors are currently approved for clinical use: vemurafenib and dabrafenib. Intracranial activity was shown for both based on two small phase II trials [157,158]; however, no study with survival as the primary endpoint was conducted initially. Most recently, a systematic examination of twenty-two studies that reported median OS data for 2153 melanoma patients concluded that treating melanoma patients who have brain metastases with BRAF^V600^ inhibitors (or immunotherapy, discussed below) may improve survival [137]. Spagnolo et al. calculated a median OS of 7.9 months for patients treated with BRAF inhibitors, relative to the historical survival of 6.2 months for patients with metastatic melanoma overall and 2.2–4.7 months for patients with intracranial metastases [137]. In addition, evidence for the importance of treating patients with effective BRAF^V600^ inhibitors has come from several reports that have determined that inhibiting BRAF^V600^ significantly prolonged the time between the initial metastatic melanoma diagnosis and the subsequent brain metastasis diagnosis [159] and also lowered the incidence of brain metastasis formation [160].

Vemurafenib was the first BRAF^V600^ inhibitor to demonstrate an increase in OS for patients as compared with dacarbazine [161], a cytotoxic DNA-alkalylating agent previously used regularly to treat metastatic melanoma. Vemurafenib has good tolerability and antitumor activity; however, early studies excluded patients with active brain metastases. More recently, OS and progression-free survival (PFS) was reported for 24 patients treated with vemurafenib who had symptomatic, non-resectable intracranial disease [162]. Dummer et al. found that 37% of patients showed a greater than 30% regression in brain metastases and 16% showed a partial response, with a median PFS of 3.9 months and median OS of 5.3 months [162]. An additional study that included 27 patients with intracranial disease treated with vemurafenib reported extracranial and intracranial response rates of 71% and 50%, respectively [163]. The median intracranial PFS was 4.6 months and median OS was 7.5 months, thus demonstrating that vemurafenib is highly active in mutant BRAF melanoma, with intracranial activity evident. These studies demonstrated an intracranial regression of melanoma metastases, suggesting that vemurafenib is at least partially able to penetrate the BBB. A small study including six patients treated with vemurafenib twice daily was performed to determine whether vemurafenib was able to cross the BBB. Sukji-Dupre et al. measured cerebrospinal fluid (CSF) concentrations of vemurafenib and compared them to plasma concentrations [164]. They reported the mean ratio of CSF/plasma concentration as only 0.98% ± 0.84%, suggesting poor penetration of the BBB by vemurafenib [164]. Additional studies with larger cohorts are needed to determine if this low level of BBB penetration is accurate. However, the intracranial response reported by Harding and Dummer suggests that either low levels of vemurafenib in the CSF are effective or that control of extracranial disease may affect intracranial melanoma metastases progression. Alternatively, establishment of brain metastases by melanoma cell transmigration through the endothelium may compromise the BBB, enabling vemurafenib to more effectively penetrate the BBB, resulting in tumor regression. These highlighted studies demonstrate the potentially therapeutic benefit of vemurafenib for patients with BRAF^V600^ brain metastases, but also reveal the critical need for additional clinical studies that are designed to examine vemurafenib treatment alone or in combination with other therapies such as MEK inhibitors (discussed below).

Dabrafenib, another BRAF^V600^ inhibitor, has also shown therapeutic benefit for melanoma patients with brain metastases. A pilot study investigated the effect of dabrafenib treatment in patients with melanoma brain metastasis and reported that 9 out of 10 patients showed brain tumor regression [165]. An additional study, with a larger cohort of 172 patients, reported a response rate of 39.2% with dabrafenib treatment of patients with the BRAF^V600E^ mutation who had no prior treatment of their brain metastases [157]. Long et al. reported a median PFS of 4.0 months and a median OS of 8.3 months [157]. A more recent analysis that focused on comparing the response of both intra- and extra-cranial metastatic melanoma to dabrafenib showed that both had high response rates and similar PFS [166]. Azer et al. corroborated the median PFS (4 months) and OS (9 months) [166] that was recorded previously by Long et al. [157]. To date, no studies have reported upon the ability of dabrafenib to penetrate the BBB in patients; however, dabrafenib distribution to the brain is also significantly limited by the BBB, though to a lesser extent than vemurafenib, as determined by in vitro transport assays and in vivo pharmacokinetic studies [167].

In a small study performed to determine why some melanoma brain metastases were poorly sensitive to vemurafenib, Harding et al. analyzed samples from 7 patients who were categorized as poorly sensitive (defined as greater than 20% tumor growth, new lesion formation, or less-than 50% tumor shrinkage for less-than 4 months). They used next-generation sequencing and found that tumors that were insensitive to vemurafenib contained co-occurring mutations that resulted in activation of the PI3K-AKT pathway [163]. This demonstrates that melanomas resistant to vemurafenib may be sensitive to combination therapies that target the PI3K-AKT pathway concomitantly with MAPK inhibition. However, only a small number of samples were analyzed and no clinical trials are currently being performed to evaluate PI3K/AKT inhibitors with or without BRAF inhibitors. However, additional clinical trials designed to test the efficacy of combination therapies that include inhibitors of the mitogen-activated protein kinase kinase (MEK), which is downstream of BRAF in the MAPK pathway, are currently underway (see Table 1). These include treatments with combinations of the MEK inhibitor, trametinib, with dabrafenib (clinical trials NCT02039947 & NCT01978236), or vemurafenib with another MEK inhibitor, cobimetinib (clinical trial NCT02537600). An additional clinical trial is assessing the therapeutic outcome of treating brain metastases with dabrafenib and SRS (clinical trial NCT01721603). As of the publication date of this manuscript, these trials are either still recruiting or are currently evaluating whether combination therapies benefit patients with BRAF^V600^ brain metastases. The results from these investigations will hopefully bring about therapeutic improvements and an increased understanding of the efficacy of inhibiting the often hyper-activated MAPK pathway with the goal of improving the survival of patients with brain metastases.

### 5.7. Immune-Based Therapies

Immune-based therapies have yielded some promising results for the treatment of melanoma brain metastasis. High-dose (HD) interleukin-2 (IL-2), a cytokine-based immunotherapy, was the first immunotherapy to be used for patients with metastatic melanoma. HD IL-2 produces durable, complete remission for a small percentage of metastatic melanoma patients (<10%) [168], but has been associated with severe toxicity and its efficacy against brain metastases is limited. A retrospective analysis of 15 stage IV melanoma patients reported that 2 with brain metastases had a complete response to HD IL-2 [169]. However, a more recent retrospective review of 7 patients with brain metastases reported progressive disease in all patients and a median OS of only 6.7 months [170].

Adoptive cell transfer (ACT) of tumor-infiltrating lymphocytes, which requires the harvesting of tumor-derived T cell lymphocytes from melanoma patients, followed by expansion (with or without genetic manipulation), and re-introduction of these cells into patients after lympho-depletion, demonstrated efficacy against melanoma brain metastases in one report [171]. Hong et al. reported that 41% (7/17) of patients who received ACT had a complete response and 35% (6/17) showed a partial response [172]. Despite these encouraging results, ACT treatment has been replaced by checkpoint inhibitors.

Checkpoint inhibitors are a new class of immune-based therapy that utilize antibodies against specific inhibitory T-cell molecules to increase the amplitude and duration of T-cell responses [173]. Currently, there are two that are approved for the treatment of melanoma metastasis: ipilimumab, a monoclonal antibody against cytotoxic T-lymphocyte-associated protein 4 (CTLA-4), and pembrolizumab or nivolumab, antibodies against programmed cell death protein 1 (PD-1) [174,175,176]. Patients with brain metastases who were treated with immune-based checkpoint blockade antibodies, reported in a systematic review of recent clinical trials that included 2153 patients, had a median OS of 7.0 months [137].

A phase II trial of ipilimumab, which included patients with symptomatic and asymptomatic brain metastases, found that 10% and 24% achieved partial response or stable disease, respectively. Median OS was 3.7 and 7 months, and 2-year OS was 10% and 24%, respectively [177]. Another phase II study was designed to evaluate ipilimumab combined with fotemustine, a chloroethyl-nitrosourea alkylating agent, for the treatment of patients with asymptomatic brain metastases. Half of all patients (10/20) showed disease control with a median PFS of 4.3 months [178]. A retrospective review by Knisely et al. assessed survival in patients who received SRS in addition to other clinical therapeutics and reported that patients who received ipilimumab as part of their therapeutic regimen had a median OS of 21.3 months compared with 4.9 months for patients without ipilimumab. The 2-year survival rates were 47% and 20%, respectively [179]. Several other studies also have reported a clinical benefit when ipilimumab was combined with other therapies, such as SRS [180,181,182,183,184,185,186,187].

Anti-PD-1 immunotherapies (pembrolizumab and nivolumab) have been evaluated for their efficacy in treating melanoma brain metastases, although the data is limited. One phase II clinical trial of patients with untreated or progressive melanoma brain metastases showed pembrolizumab activity in brain metastases with 4 of 18 patients responding to treatment. The drug also demonstrated an acceptable safety profile [188]. In a second phase II clinical trial of pembrolizumab for patients with asymptomatic melanoma brain metastasis, the drug was found once again to possess brain activity, with 4 of 14 patients exhibiting a partial response and progression-free survival in the range of 6 to 17 months [189]. Nivolumab likewise, appears to show activity against melanoma brain metastases. A retrospective study designed to analyze nivolumab efficacy in combination with SRS in patients with melanoma brain metastases reported a median overall survival of 12 months. The authors concluded that disease control and OS appeared to be extended for patients on nivolumab compared with patients on conventional therapies [190].

Although the benefits of recent therapeutic breakthroughs have improved clinical outcomes for patients with melanoma brain metastasis [135], substantial advances in this patient population remain elusive. Additional clinical trials will assess the efficacy of immunotherapies alone or in combination with other therapeutic intervention strategies against melanoma brain metastases. Several studies are underway including: NCT01703507, NCT02085070, NCT02097732, NCT02320058, NCT02460068, NCT02374242, NCT02662725, NCT02681549, NCT02621515, NCT02716948, and NCT02107755 (Table 1), the results of which will enhance our understanding of immunotherapy intervention as it relates to brain metastases and ultimately improve patient care.

## 6. Conclusions

Studies aimed at better understanding chemokine signaling, cell arrest in the brain microvasculature, extravasation, and vascular co-option have brought to light important molecules that may promote brain tropism and potentially reveal novel targets for therapeutic intervention. Many of the recently identified mechanisms exhibit links to the PI3K/AKT signaling pathway and in vitro and in vivo studies of PI3K/AKT pathway inhibition for brain metastatic melanoma have already begun. Using a pan-class I PI3K inhibitor (buparlisib), Niessner et al. demonstrated diminished AKT activity, decreased cell growth and proliferation, and increased apoptosis in numerous metastatic melanoma cell lines with various mutational profiles. Buparlisib is also able to cross the BBB and inhibited growth of melanoma brain metastases in nude mice [107]. Although these results are encouraging, many additional tests are required before buparlisib or other PI3K/AKT inhibitors can advance to clinical trials. A more thorough understanding of the role of the PI3K/AKT pathway as well as other signaling pathways in melanoma brain metastasis biology is still needed. Melanoma has the highest somatic mutational load of all solid tumor types [191], which makes understanding the intricate mechanisms of oncogenicity extremely complex. In addition, extrinsic factors contribute to melanoma brain tropism, such as the influence of the BBB and brain microenvironment on melanoma cell homing, extravasation, and genetic reprogramming. These complexities, combined with the difficulty of creating pharmaceuticals that are safe, effective, specific, and able to penetrate the BBB make the mission to develop improved treatments for these patients extremely difficult. Animal models of melanoma brain metastasis have become an integral component in the quest to overcome many of these challenges as they offer a conduit through which important discoveries may be translated to the clinic. As meaningful new discoveries are made, in vivo models will be used to validate findings. Modern targeted therapies and immunotherapies will continue to be tested in vivo and later tested in a broader range of more sophisticated and relevant animal models for efficacy against brain metastases; future therapeutic strategies based on current basic research will follow suit—all with the goal of improving melanoma patient survival.

## Figures and Tables

**Table 1:** Summary of clinical trials in progress for patients with metastatic melanoma ^1^.

Trial	Phase	Status	Primary Outcome Measured	Title
NCT01721603	II	Active, not recruiting	Safety/efficacy	A Phase 2 Prospective Trial of Dabrafenib with Stereotactic Radiosurgery in BRAF^V600E^ Melanoma Brain Metastases
NCT02115139	II	Recruiting	1-year survival rate	GEM STUDY: Radiation and Yervoy in Patients with Melanoma and Brain Metastases (GRAY-B)
NCT01703507	I	Active, not recruiting	Maximum tolerated dose ipilimumab	Phase I Study of Ipilimumab Combined with Whole Brain Radiation Therapy or Radiosurgery for Melanoma
NCT02085070	II	Recruiting	Response rate	MK-3475 in Melanoma and NSCLC Patients with Brain Metastases
NCT02097732	II	Recruiting	Local control rate	Ipilimumab Induction in Patients with Melanoma Brain Metastases Receiving Stereotactic Radiosurgery
NCT02039947	II	Recruiting	Intracranial response rate	Study to Evaluate Treatment of Dabrafenib Plus Trametinib in Subjects with BRAF Mutation-Positive Melanoma That Has Metastasized to the Brain
NCT01378975	II	Completed, no posts	Best overall response rate	A Study of Vemurafenib in Metastatic Melanoma Patients with Brain Metastases
NCT01978236	II	Recruiting	Concentrations of dabrafenib & trametinib in metastases	Dabrafenib/Trametinib, BRAF or BRAF AND MEK Pre-op with BRAF and MEK Post-op, Phase IIB, Melanoma with Brain Mets, Biomarkers and Metabolites
NCT02320058	II	Recruiting	Clinical benefit rate	A Multi-Center Phase 2 Open-Label Study to Evaluate Safety and Efficacy in Subjects with Melanoma Metastatic to the Brain Treated with Nivolumab in Combination with Ipilimumab Followed by Nivolumab Monotherapy (CheckMate 204)
NCT01503827	III	Recruiting	Distant intracranial failure	Whole Brain Radiotherapy Following Local Treatment of Intracranial Metastases of Melanoma (WBRTMel)
NCT01644591	III	Active, not recruiting	Time to local failure	Trial to Compare Local Control and Neurocognitive Preservation after Initial Treatment with Stereotactic Radiosurgery (SRS) versus Whole Brain Radiation Therapy (WBRT) for Patients with >3 Brain Metastases from Melanoma
NCT02460068	III	Recruiting	Overall survival rate	A Study of Fotemustine(FTM) vs. FTM and Ipilimumab (IPI) or IPI and Nivolumab in Melanoma Brain Metastasis (NIBIT-M2)
NCT02374242	II	Recruiting	Intracranial response rate	Anti-PD 1 Brain Collaboration for Patients with Melanoma Brain Metastases (ABC)
NCT02662725	II	Completed, no posts	Overall survival rate	Ipilimumab Combined with a Stereotactic Radiosurgery in Melanoma Patients with Brain Metastases (IPI + RTS)
NCT02308020	II	Recruiting	Complete response, partial response, objective intracranial response rates	A Study of Abemaciclib (LY2835219) in Participants with Breast Cancer, Non-small Cell Lung Cancer, or Melanoma That Has Spread to the Brain
NCT02681549	II	Recruiting	Brain metastasis response rate	Pembrolizumab Plus Bevacizumab for Treatment of Brain Metastases in Metastatic Melanoma or Non-small Cell Lung Cancer
NCT02621515	II	Recruiting	Best overall response rate	Nivolumab in Symptomatic Brain Metastases (CA209-322)
NCT02716948	I	Recruiting	Incidence of serious adverse events	Stereotactic Radiosurgery and Nivolumab in Treating Patients with Newly Diagnosed Melanoma Metastases in the Brain or Spine
NCT01904123	I	Not yet recruiting	Maximum tolerated dose WP1066	A Phase I Trial of WP1066 in Patients with Central Nervous System (CNS) Melanoma and Recurrent Glioblastoma Multiforme (GBM)
NCT02452294	II	Recruting	Intracranial disease control rate	Buparlisib in Melanoma Patients Suffering from Brain Metastases (BUMPER)
NCT02537600	II	Recruiting	Complete or partial intracranial response rate	Vemurafenib and Cobimetinib Combination in BRAF Mutated Melanoma with Brain Metastasis (CONVERCE)
NCT02107755	II	Recruiting	Progression-free survival rate	Stereotactic Radiation Therapy and Ipilimumab in Treating Patients with Metastatic Melanoma
NCT01983124	II	Completed, no posts	Progression-free survival rate	Vemurafenib + Fotemustine to Treat Advanced Melanoma Patients with V600BRAF Mutation Recurred While on Vemurafenib (BeyPro1)

^1^ Trials either focus on melanoma brain metastasis or do not exclude patients with melanoma brain metastasis.
